# AI-driven molecular generation of not-patented pharmaceutical compounds using world open patent data

**DOI:** 10.1186/s13321-023-00791-z

**Published:** 2023-12-13

**Authors:** Yugo Shimizu, Masateru Ohta, Shoichi Ishida, Kei Terayama, Masanori Osawa, Teruki Honma, Kazuyoshi Ikeda

**Affiliations:** 1 HPC- and AI-driven Drug Development Platform Division, RIKEN Center for Computational Science, 1-7-22 Suehiro-cho, Tsurumi-ku, Yokohama City, Kanagawa 230-0045 Japan; 2 Division of Physics for Life Functions, Keio University Faculty of Pharmacy, 1-5-30 Shibakoen, Minato-ku, Tokyo, 105-8512 Japan; 3 Graduate School of Medical Life Science, Yokohama City University, 1-7-29 Suehiro-cho, Tsurumi-ku, Yokohama City, Kanagawa 230-0045 Japan; 4 RIKEN Center for Biosystems Dynamics Research, 1-7-22 Suehiro-cho, Tsurumi-ku, Yokohama City, Kanagawa 230-0045 Japan

**Keywords:** Patented compounds, Drug discovery, Database, Compound search, Molecular generation, Reward function

## Abstract

Developing compounds with novel structures is important for the production of new drugs. From an intellectual perspective, confirming the patent status of newly developed compounds is essential, particularly for pharmaceutical companies. The generation of a large number of compounds has been made possible because of the recent advances in artificial intelligence (AI). However, confirming the patent status of these generated molecules has been a challenge because there are no free and easy-to-use tools that can be used to determine the novelty of the generated compounds in terms of patents in a timely manner; additionally, there are no appropriate reference databases for pharmaceutical patents in the world. In this study, two public databases, SureChEMBL and Google Patents Public Datasets, were used to create a reference database of drug-related patented compounds using international patent classification. An exact structure search system was constructed using InChIKey and a relational database system to rapidly search for compounds in the reference database. Because drug-related patented compounds are a good source for generative AI to learn useful chemical structures, they were used as the training data. Furthermore, molecule generation was successfully directed by increasing and decreasing the number of generated patented compounds through incorporation of patent status (i.e., patented or not) into learning. The use of patent status enabled generation of novel molecules with high drug-likeness. The generation using generative AI with patent information would help efficiently propose novel compounds in terms of pharmaceutical patents. Scientific contribution: In this study, a new molecule-generation method that takes into account the patent status of molecules, which has rarely been considered but is an important feature in drug discovery, was developed. The method enables the generation of novel molecules based on pharmaceutical patents with high drug-likeness and will help in the efficient development of effective drug compounds.

**Supplementary Information:**

The online version contains supplementary material available at 10.1186/s13321-023-00791-z.

## Introduction

Generative artificial intelligence (AI) is an aspect of AI application that has received significant attention, particularly, as an important tool for drug discovery; consequently, numerous chemical structure-generating AIs have been reported [1, 2]. Development of the methods implemented by these AIs to learn and generate chemical structures is one of the ways in which they are currently evolving; various methods have been proposed, including generative adversarial networks [3, 4], recurrent neural networks (RNN) [5, 6], and transformers [7, 8]. The implementation of techniques, such as genetic algorithms [9], variational autoencoders [10], and reinforcement learning [6, 11, 12], to produce molecules with desired properties is another way in which chemical structure-generating AIs are evolving. The inclusion of properties, such as pharmaceutical activity and absorption, distribution, metabolism, excretion, and toxicity (ADMET), are important during drug discovery. For example, potentially active compounds or compounds with desirable ADMET properties can be generated based on their prediction scores of machine learning or deep learning models using features, such as molecular properties, fingerprints, and graph descriptors, and docking scores obtained by molecular docking against target protein structures. In addition, generative AI that can simultaneously optimize multiple properties has been reported [13, 14].

Although patent information is an important source in drug discovery, it is rarely considered in structure-generating AI. Using generative AI trained with patented compound data of tyrosine kinase inhibitors, Subramanian et al. [15] generated molecules structurally similar to FDA-approved drugs, such as erlotinib, by calculating their Tanimoto similarities as the property to be optimized. However, it is not known if the generated molecules are patented. Obtaining intellectual property rights, particularly substance patents, is important in drug discovery to protect the discovered molecules. Despite its importance, patentability is also rarely considered when using generative AI. This is likely because validating patentability requires specialized equipment, such as the use of patent-specific commercial software and databases [16]; additionally, automatically authenticating patentability through calculations or other means is difficult.

The recent availability of open patent data, such as patent documents provided by Google [17] and patent–compound information provided by SureChEMBL [18], has enabled the development of unprecedented approaches to the patenting of compounds. Attempts have been made to extract patents related to drug discovery. Falaguera et al. [19] used patent classification, and Subramanian et al. [15] used keyword searches to extract patents related to drug discovery from patents published by the United States Patent and Trademark Office (USPTO). However, validating the patentability of compounds in drug discovery requires a global approach; therefore, using only the USPTO patents is insufficient.

This study aimed to create a generative AI that can use information on the global patentability of generated molecules to direct exploration of chemical space of the molecules. To this end, chemical structures included in pharmaceutical-related patent documents published in the world were collected from open patent sources and incorporated into a drug-related patented compound database. To generate novel molecules by exploring and expanding chemical space of patentable compounds in drug discovery, calculation system of properties, which represent the patentable status of generated molecules, were developed. The properties were computed in the form of reward functions and learned in a generative AI.

## Materials and methods

### Data preparation and integration for drug-patent database

Drug-related patented compounds were collected to develop a reward function that can determine if a generated molecule is present in drug-related patents. The SureChEMBL database (January 2021) was used as the source of drug-related patented compounds because it consists of 20,000,411 compounds from 4,799,617 patents, covering patent authorities, the World Intellectual Property Organization, and the patent offices of Europe, the United States, and Japan.

The extraction of only drug-related patented compounds from the SureChEMBL compounds was necessary because that database contains drug-related and non-drug-related patented compounds (e.g., food, fertilizers, dyes, oils, and organic compounds). To extract drug-related patented compounds, two types of patent classification information were used: International Patent Classification (IPC) and Cooperative Patent Classification (CPC). Patents classified as A61K (preparations for medicinal, dental, or toilet purposes) or A61P (specific therapeutic activity of chemical compounds or medicinal preparations) were defined as drug-related. Patent numbers and compounds described in patents were extracted from the SureChEMBL downloadable bulk dataset. Because the downloadable dataset did not contain IPC/CPC information, patent numbers and their IPC/CPC codes were extracted from Google Patents Public Datasets [20]. The IPC/CPC information was then attached to SureChEMBL based on their patent numbers, resulting in 13,448,634 compounds in 1,057,881 drug-related patents. The SQL codes used to retrieve the IPC/CPC information from Google Patents Public Datasets are available in Additional file 1: Method S1.

Chemical structures registered in SureChEMBL that were erroneous or possibly erroneous were removed. A part of compounds in SureChEMBL are registered by the automated chemical entity recognition of images of the chemical structures in patent documents. The registered chemical structures that were identified using optical character recognition (OCR) are denoted as OCR structures. Structural errors were observed in some OCR structures; for instance, SCHEMBL13574165, registered as a compound in patent WO-2009153592-A1, has an incorrect structure. The original compound has a pyrazole ring; however, the ring is broken in the SureChEMBL entry (Additional file 1: Fig. S1). A recognition error during the conversion of the structural image to the chemical structure likely caused the registration of incorrect structures. Although some OCR structures were correctly recognized, verifying the accuracy of all OCR structures was difficult; therefore, all OCR structures—2,727,799 structures that are annotated as appearing in compound images of patent documents—were removed from the study to prevent the incorporation of these inaccuracies. This resulted in a reduction of the number of drug-related patented compounds from 13,448,634 to 10,720,835.

**Fig. 1:**
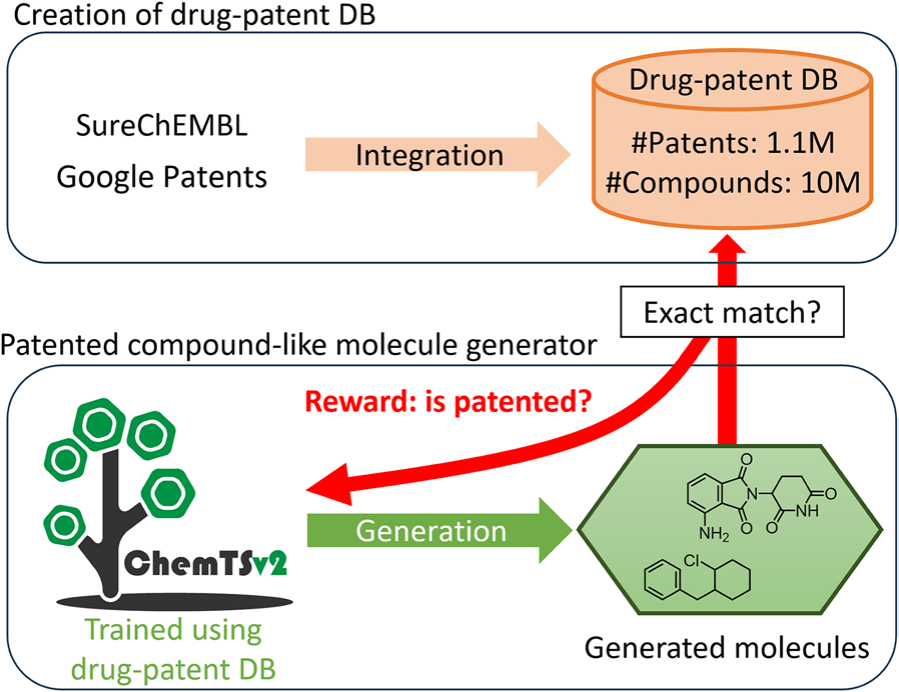
Overview of this study

### Creation of drug-related patented compound database

To implement the reward function, which determines if the generated structure was included in the 10,720,835 drug-related patented compounds, a relational database of drug-related patented compounds (drug-patent DB) and a search system to examine the generated structure were created (Fig. 1). The chemical structures of 10,720,835 compounds were standardized and desalted using ChEMBL Structure Pipeline 1.0.0 [21]. InChIKeys [22], 27-character strings representing the chemical structures, were generated without stereochemical layer using RDKit 2022.03.2 [23] and stored in the drug-patent DB using an SQLite 3.36.0 library to be used in text-based search systems. The InChIKey index was created for a rapid search. The drug-patent DB includes the chemical structures of drug-related patented compounds in the form of InChIKeys and their SureChEMBL entry identifiers, which are easily connected to the SureChEMBL information (e.g., the original chemical structures and patent numbers).

### Preparation of training data for RNN

ChemTSv2 software was used for chemical structure generation because it can easily incorporate user-defined reward functions [24]. ChemTS generates molecular structures using an RNN trained on SMILES [25] strings and explores structures using Monte Carlo tree search (MCTS) [26] with the desired properties defined as a reward function [12, 13]. Structures in databases, such as ChEMBL [27] or ZINC [28], can be used as learning sources for RNN; however, the structures of drug-related patented compounds were used to learn a more specific RNN for patented compounds (patent RNN) in this study. Because 10,720,835 compounds were surplus to the requirement for RNN training, approximately 250,000 compounds were selected for this purpose. The training data for the patent RNN were extracted from the drug-patent DB using the following procedure: First, five million compounds were randomly selected from the DB. Subsequently, compounds that were not appropriate for training, such as those lacking SMILES, having no ring, and containing non-drug-like elements (e.g., metals and isotopes) and substructures (Additional file 2), were removed. Compounds consisting of multiple components were then desalted using the KNIME RDKit, leaving only molecules of one component. Thereafter, atypical compounds, belonging to both ends of the distribution of molecular properties, such as the number of atoms, number of heavy atoms, molecular weight, SlogP, number of aromatic rings, and fraction of sp^3^ carbon atoms, were removed, resulting in a remaining selection of compounds (4,008,514 molecules including 1,177,174 unique Murcko scaffolds). From these compounds, 247,738 molecules were randomly selected. The selected molecules included 145,443 unique Murcko scaffolds. Finally, the structures were standardized using the ChEMBL Structure Pipeline and converted into canonical SMILES strings (RDKit) to be used as training data for the patent RNN model. The training data for RNN are available in Additional file 3.

### RNN training and parameter optimization

To train the patent RNN model, four parameters—dropout rate, learning rate, batch size, and number of hidden state dimensions of gated recurrent units—were optimized using Optuna [29] to maximize the ratio of the chemically interpretable, filter-passed, and unique SMILES strings to all SMILES strings generated in 15 min of molecular generation (see “Generation of molecules using ChemTS” section for details of the filters used). The remaining parameters for training were set to default, excluding the length of the sequence for padding the SMILES tokens, which was the maximum token length (109) of the training data. Training was performed for 500 epochs, with 10% of the data being used for validation. Molecular generation during parameter optimization was performed using a reward function that always returned a value of one to eliminate the influence of the reward function; herein, *C* was set to 1.0, and the other parameters to the default values. The optimization resulted in dropout rate, learning rate, batch size, and units of 0.1077, 0.000434, 384, and 896, respectively.

### Reward functions

To assess patentability when generating molecules, a method that determines if the generated compounds were included in drug-related patented compounds was used. Two reward functions (*R*_patent_ and *R*_not-patent_), which yield opposing results, were defined. For a given generated molecule *x*, the reward *R*_patent_ is defined as:

$${R}_{\text{patent}}\;(x)= \left\{\begin{array}{ll}1 &\text{if}\;x\;\text{is}\;\text{matched}\;\text{to}\;\text{a}\;\text{patented}\;\text{compound}\;\text{in}\;\text{the}\;\text{drug-patent}\;\text{DB}\\ 0 & \text{if}\;x\;\text{is}\;\text{not}\;\text{matched}\;\text{to}\;\text{any} \;\text{patented}\;\text{compounds}\;\text{in}\;\text{the}\;\text{DB}\end{array}\right.$$

and the reward *R*_not-patent_ is defined as:

$${R}_{\text{not-patent}}\;(x)= \left\{\begin{array}{ll}1 & \text{if}\;x\;\text{is}\; \text{not}\; \text{matched}\; \text{to}\; \text{any}\; \text{patented}\; \text{compounds}\; \text{in}\; \text{the}\; \text{DB}\\ 0 & \text{if}\;x\;\text{is}\; \text{matched}\; \text{to}\; \text{a}\; \text{patented}\; \text{compound}\; \text{in}\; \text{the}\; \text{DB} \end{array}\right.$$

When *R*_patent_ is used as the ChemTS reward function, ChemTS tries to generate molecules that can be found in drug-related patents; when *R*_not-patent_ is used, ChemTS tries to generate molecules that are not found in drug-related patents. To test the baseline performance of the models, a random reward function *R*_rand_, which returns a random value between zero and one, was used.

### Methods used to identify an exact match between two compounds

Two types of methods—fingerprint-based and text-based—were used to identify generated molecules that can also be found in the drug-patent DB. These methods were implemented through Python scripts using RDKit. In the fingerprint-based method, the drug-patent DB compounds were converted into MinHash fingerprints (MHFP6, 2048 bits) [30]. The locality-sensitive hashing (LSH) approximate nearest neighbor search using the MHFP6 with LSHForestHelper [31] was used to examine the exact match between query compounds and the drug-patent DB compounds. As a baseline, a fingerprint-based search method using Morgan fingerprints (radius = 3, 2048 bits) with BulkTanimotoSimilarity function of RDKit was also used. In the text-based method, InChIKeys of the two compounds were compared to determine if they were exact matches. Query molecules were searched against drug-patent DB using the SQL SELECT command in the Python sqlite3 module.

### Validity and uniqueness of molecular generation

The validity and uniqueness of molecular generation were calculated as follows:

$$\text{Validity}=\frac{\text{Number}\; \text{of}\; \text{valid}\; \text{generated}\; \text{SMILES}\; \text{strings}}{\text{Number}\; \text{of}\; \text{all}\; \text{generated}\; \text{SMILES}\; \text{strings}}$$

$$\text{Uniqueness}=\frac{\text{Number}\; \text{of}\; \text{unique}\; \text{generated}\; \text{SMILES}\; \text{strings}}{\text{Number}\; \text{of}\; \text{all}\; \text{valid}\; \text{generated}\; \text{SMILES}\; \text{strings}}$$

where a valid SMILES string indicates that the generated SMILES string is interpretable as a molecule by RDKit and is not filtered out by ChemTS filters. Notably, this “validity” is a combination of “validity” and “filters” defined in the benchmarks of molecular generation models, such as MOSES [32] and Guacamol [33].

### Generation of molecules using ChemTS

By adjusting the value of the MCTS exploration parameter *C* of ChemTS, the user can control the way of search; particularly, the user can decide to search deeper into the chemical space around previously generated structures or perform a shallower search in a different space. Molecular generations using a large *C* (e.g., 1.0) explore new chemical spaces more extensively, whereas those using a small *C* (e.g., 0.1) explore narrower chemical spaces more deeply. Six values of *C*—0.1, 0.2, 0.4, 0.6, 0.8, and 1.0—were used in this study. The ChemTS user can remove undesired generated molecules using prepared or user-defined filters in various settings. In this study, at each generation step, a generated SMILES string was removed if it was trapped by at least one of five ChemTS filters that filter out (1) molecules with rarely occurring molecular patterns based on the frequency of occurrence in the PubChem database [12, 34], (2) those that violate at least one of Lipinski’s rule of five [35], (3) those that contain radicals, (4) those with a synthetic accessibility score [36] ≥ 3.5, and (5) those with a ring size > 6. Molecules removed by the filters were not used in reward calculations. At each *C* setting, molecular generation was performed in triplicate. Each run was executed for 24 h using a GPU (Nvidia Quadro RTX 8000) and at least 250,000 valid and unique molecules were generated.

### Visualization of chemical space

The chemical space of the generated molecules was visualized using a uniform manifold approximation and projection (UMAP) that projects close points in a high-dimensional space onto close points in a low-dimensional space [37]. UMAP-learn 0.5.3 was used [38] with a Jaccard metric to transform the 2048-bit Morgan fingerprint (radius = 2) arrays of compounds into two-dimensional components (UMAP components 1 and 2). To show the chemical space of patented pharmaceutical compounds, 500,000 molecules randomly selected from the drug-patent DB were included in the calculation.

### Analysis of generated molecules

Structural similarities between the generated molecules and the molecules in the drug-patent DB were calculated as the Tanimoto coefficient of Morgan fingerprints (radius = 2, 2048 bits) using RDKit. The quantitative estimate of drug-likeness (QED) [39] values of the generated molecules were calculated using RDKit.

## Results and discussion

### Selection of a method that can identify an exact match

**Fig. 2:**
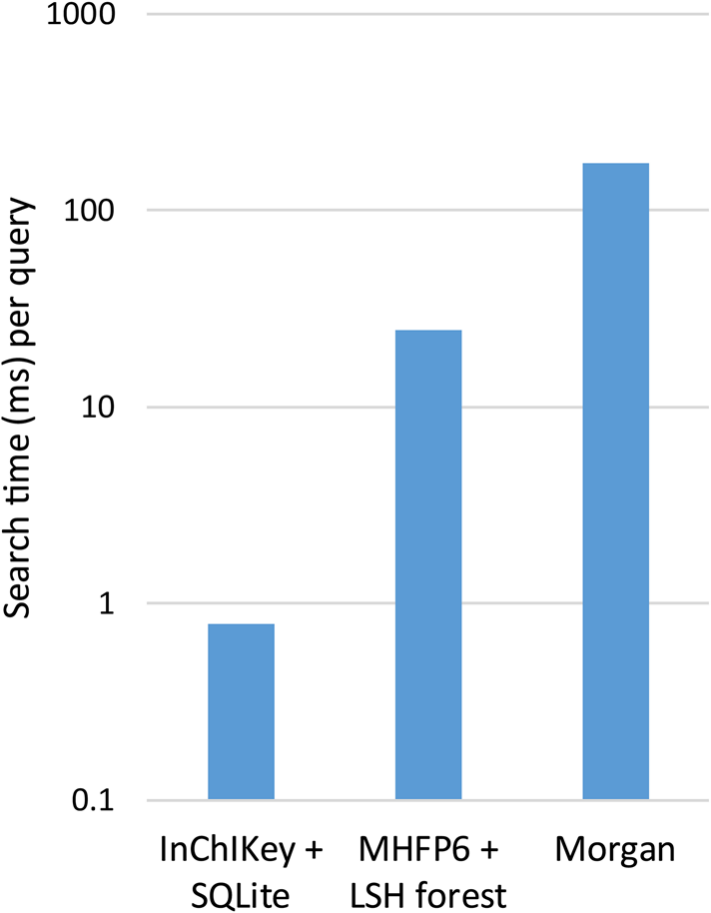
Calculation times for the identification of exact matches using three methods. Searches for 10,000 query compounds were performed against 1,000,000 drug-related patented compounds. The search time is shown in the log scale

The computation times for the matching of 10,000 molecules randomly selected from SureChEMBL with 1,000,000 molecules randomly selected from the drug-patent DB using the Morgan, MHFP6, and InChIKey methods were 3.8 h, 4.1 min, and 7.9 s, respectively (Fig. 2). The time required for preparing the reference dataset, such as creating the LSH forest, SQL database, and SQL index, was excluded from the computation time. Text-based InChIKey was the fastest method, matching the compounds approximately 1733 times faster than the Morgan fingerprint-based method. The speed of the text-based InChlKey was sufficient for practical use in ChemTS. Although the MHFP6 method was approximately seven times faster than the Morgan fingerprint method, it was slower than the InChlKey method and required a large amount of memory. Therefore, the InChIKey method was used in the reward function to identify exact matches between two compounds.

### Creation of patent RNN models for molecular generation

After optimizing the ChemTS hyperparameters for training, the optimized patent RNN model generated molecules containing 47.5% filter-valid and unique SMILES strings within 15 min. In comparison, molecular generation was performed using the ChemTS ZINC RNN model trained with commercial compounds extracted from the ZINC database. The filter-valid and unique SMILES rate was 38.2% for the ChemTS ZINC model, suggesting that the patent RNN model could generate valid molecules more efficiently than the ChemTS ZINC model. A detailed analysis of the performance of AIs generating a large number of molecules is described in the following section.

### Molecular generations using the patent RNN model

To evaluate the ability to generate molecules independent of the reward function, the validity and uniqueness of ChemTS using the patent RNN model with a random reward function *R*_rand_ in a *C* = 1.0 setting, were assessed. The first 250,000 valid and unique molecules generated using the patent RNN were compared with those generated using the ZINC RNN model. The average validity and uniqueness of the patent RNN model were 0.45 and 0.84, respectively; those of the ZINC RNN model were 0.39 and 0.92, respectively. These results showed that the performances of the patent RNN model was comparable to that of the ChemTS ZINC RNN model for the generation of valid and unique molecules.

**Fig. 3:**
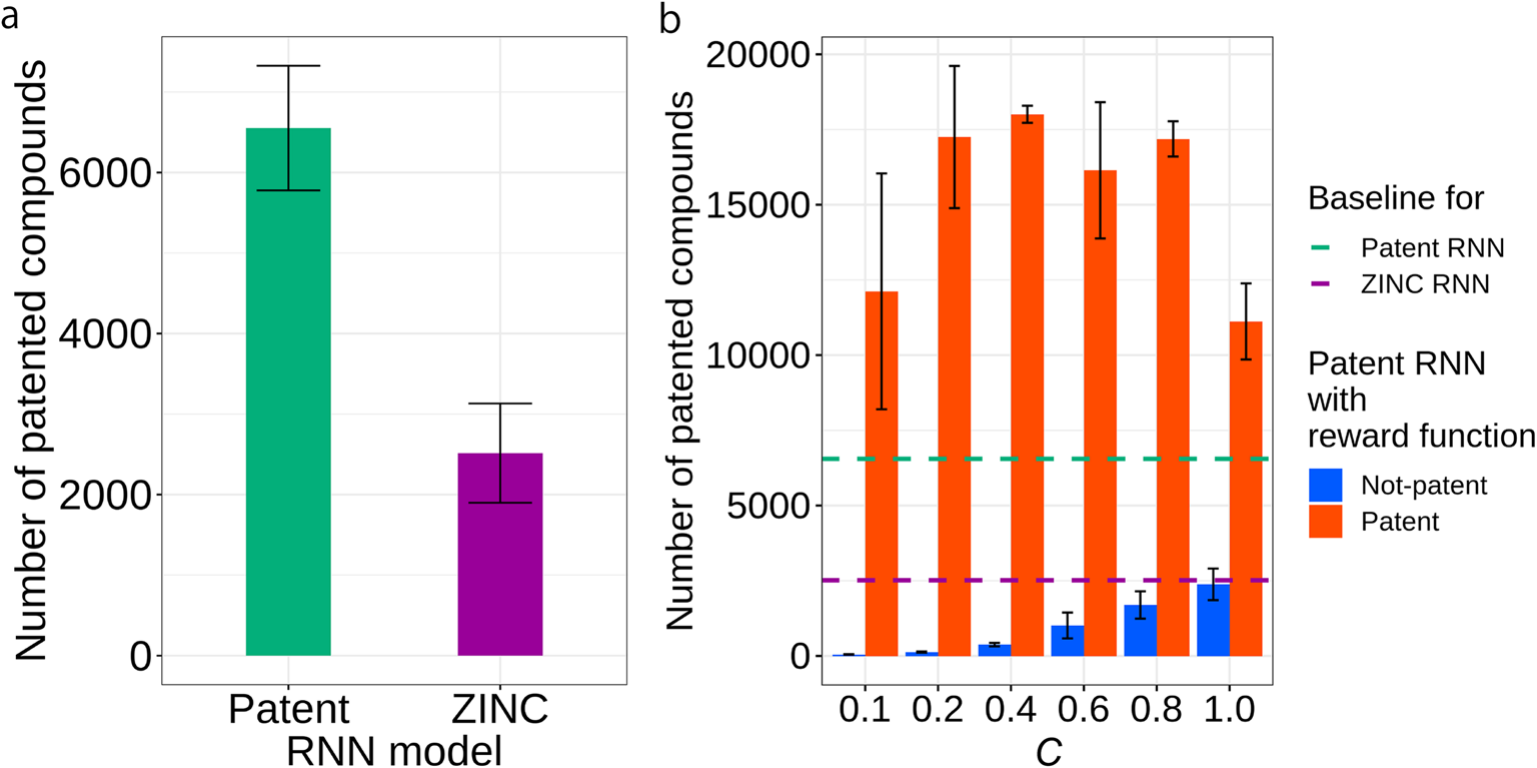
Number of generated molecules matched to the drug-patent DB compounds. Each bar represents the average values of three replicate molecular generations. Error bars represent the standard deviation. In each run, the first 250,000 valid and unique molecules that were generated were evaluated. **a** Number of patented compounds generated by the patent RNN model and the ChemTS ZINC RNN model under conditions: *C* = 1.0 and *R*_rand_ reward function. These numbers are used as baseline values of patented compound-generations for the RNN models. **b** Number of patented compounds generated by the patent RNN model across varying reward functions. The baseline values of the patent/ZINC RNN models are also shown as dashed lines

The patented-compound-generating ability of the patent RNN model was evaluated. The number of patented compounds generated by the patent RNN model that could be found in the drug-patent DB was 2.6-fold higher than those generated by the ZINC model (Fig. 3a). This result indicates that the patent RNN model was better suited for generating drug-related patented compounds. However, the drug-related patented compounds generated by the patent RNN model represented a small percentage (2.6%) of the 250,000 molecules.

### Reward functions

The effect of reward functions on the generation of drug-patent DB molecules were examined by comparing the number of drug-patent DB molecules generated by the RNN model using the *R*_patent_ reward function with that of the RNN model using *R*_not-patent_. Under all conditions of *C*, the number of drug-related patented compounds generated using *R*_patent_ was higher than that generated by *R*_not-patent_ (Fig. 3b). Furthermore, the number of patented compounds generated using *R*_patent_ was higher than that generated by the random reward function *R*_rand_; additionally, the number of patented compounds generated by *R*_not-patent_ was lower than that generated by *R*_rand_, indicating that *R*_patent_ and *R*_not-patent_ performed as intended. When *R*_not-patent_ was used as the reward function, the number of generated drug-related patented compounds increased as the value of *C* increased. The larger the value of *C*, the larger the variety of scaffolds generated, and the more likely the compounds generated using *R*_not-patent_ are going to be patented.

### Chemical space of generated molecules

**Fig. 4:**
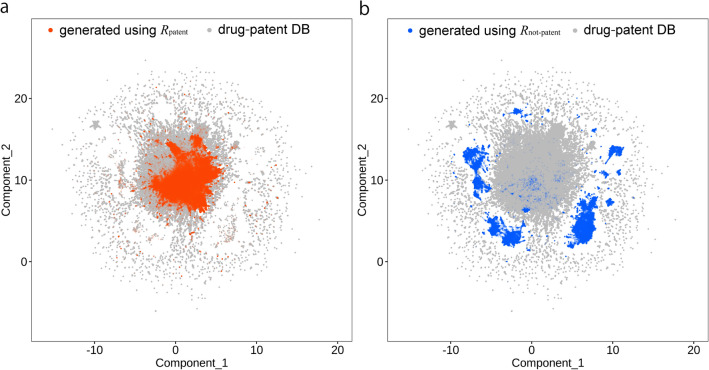
Chemical space of generated molecules and the drug-patent DB compounds. Molecules generated using *R*_patent_ and *R*_not-patent_ as reward functions in a *C* = 0.1 setting were compared with 500,000 drug-patent DB compounds. The generated molecules using the *R*_patent_ and *R*_not-patent_ rewards were shown in orange (**a**) and blue (**b**), respectively, and the drug-patent DB compounds were colored gray in the background. The chemical space was visualized using UMAP

With regard to the structural fingerprints, the chemical space of the molecules generated using *R*_patent_ and *R*_not-patent_ was compared with that of the compounds in the drug-patent DB. The chemical space of most molecules generated using *R*_patent_ was distributed within that of the drug-patent DB compounds, particularly in the region indicated by the dense gray dots where the drug-patent DB compounds were abundant (Fig. 4a and Additional file 1: Fig. S2). Therefore, although the 250,000 molecules generated using *R*_patent_ do not cover the entire chemical space of the drug-patent DB compounds, molecules corresponding to a significant proportion of the space were generated. Collectively, the results shown in Figs. 3b and 4a indicate that most of the 250,000 compounds generated using *R*_patent_ were in the chemical space of the drug-patent DB compounds; additionally, more than 10,000 of the generated compounds matched the 10,720,835 drug-patent DB compounds, regardless of the value of *C*. However, most of the molecules generated using *R*_not-patent_ and *C* = 0.1 were distributed in the region that was not occupied by the drug-patent DB compounds (Fig. 4b). Combining the results of Figs. 3b and 4b indicates that most of the 250,000 compounds generated by *R*_not-patent_ and *C* = 0.1 were in the least populated regions of the chemical space of the drug-patent DB compounds; therefore, less than 3000 compounds produced using this method matched the drug-patent DB compounds. On the other hand, in the *C* = 0.2–1.0 settings, most of the molecules generated using *R*_not-patent_ were distributed in the region that was occupied by the drug-patent DB compounds (Additional file 1: Fig. S2). In addition, in high values of *C* (0.8 and 1.0), the molecules generated using *R*_not-patent_ were distributed in the same region to that was occupied by the molecules generated using *R*_patent_; therefore, relatively large number of patented molecules were found in the molecules generated using *R*_not-patent_.

### Novelty of generated molecules

**Fig. 5:**
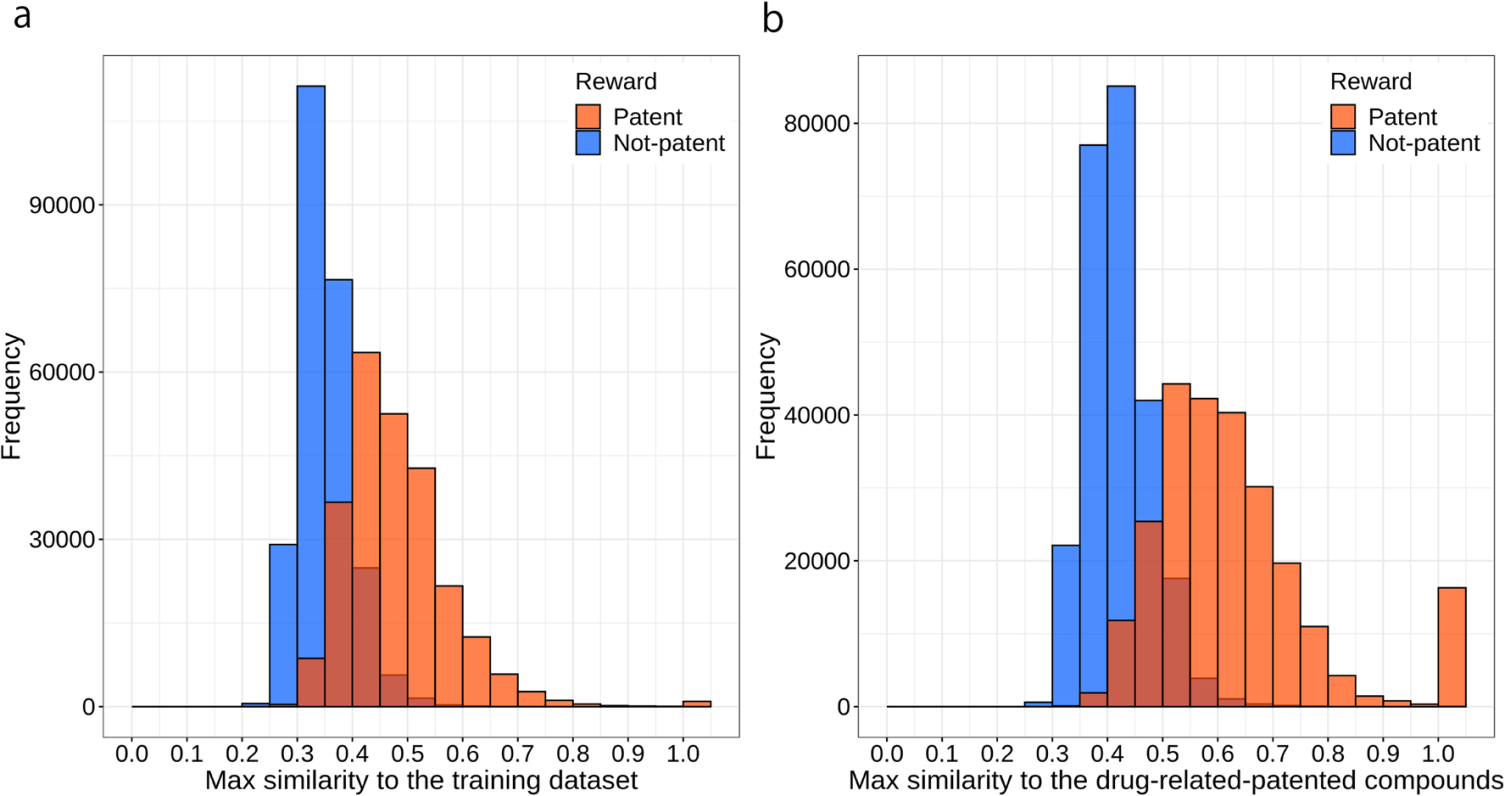
Frequency of structural similarities of generated molecules against the drug-patent DB compounds. **a** Maximum similarities of generated molecules using the *R*_patent_ (orange) and *R*_not-patent_ (blue) rewards to the 247,738 drug-patent DB compounds used as training data of the patent RNN. **b** Maximum similarities of generated molecules using the *R*_patent_ (orange) and *R*_not-patent_ (blue) rewards to the drug-patent DB compounds. Molecular generation was performed at *C* = 0.1

ChemTS generated 250,000 molecules using the patent RNN model, *R*_patent_ reward function, and *C* = 0.1; however, 946 molecules (0.38%) matched (i.e., similarity = 1) the training data compounds (247,738 drug-patent DB compounds) for the patent RNN model (Fig. 5a). Thus, 99.6% of the generated molecules were not included in the training data. The peak of the distribution was where the similarity to the training compounds was between 0.4 and 0.45. Molecules similar to the training compounds—similarity of 0.7 or greater—were also generated at a percentage of 2.2%. In the other values of *C* (0.2–1.0), similar results were observed (Additional file 1: Fig. S3). When using the *R*_not-patent_ reward in *C* = 0.1 setting, the peak of the distribution was where the similarity to the training compounds was between 0.3 and 0.35; over 99.9% of the generated molecules were similarity of < 0.7 to the training compounds (Fig. 5a). Using larger value of *C* with the *R*_not-patent_ reward, the peak of the distribution became relatively high (Additional file 1: Fig. S3). In all *C* settings, the peak of the distribution using the *R*_not-patent_ reward located in lower similarity value than that using the *R*_patent_ reward did. Regarding the maximum similarity between the 10,720,835 drug-patent DB compounds and the molecules generated by ChemTS, the peak of the distribution was where the similarity to the drug-patent DB compounds was between 0.5 and 0.55 for *R*_patent_ and between 0.4 and 0.45 for *R*_not-patent_ in the *C* = 0.1 setting (Fig. 5b). Regarding *R*_patent_, 21.5% of the molecules had a similarity ≥ 0.7, whereas only 0.1% of the molecules generated using *R*_not−patent_ had a similarity ≥ 0.7. The differences in the similarity peaks and in the rate of generation of molecules that are similar to the drug-patent DB compounds are because of the differences in reward functions. The fact that the use of *R*_patent_ in the reward function generates molecules that do not match the drug-patent DB compounds but have a very high degree of similarity suggests that it can generate molecules that are not patented but may have the desired activity.

### Drug-likeness of generated molecules

The QED for the most molecules generated with the *R*_patent_ in all *C* settings and the *R*_not-patent_ in most *C* settings was high (Additional file 1: Fig. S4). The QED for the molecules generated with the *R*_patent_ reward function was distributed in a region of higher values than that for the molecules generated using *R*_not-patent_ in each *C* setting. The difference of QED distributions using *R*_patent_ and *R*_not-patent_ was large particularly in low *C* settings (*C* = 0.1 and 0.2). Using the *R*_not-patent_ reward in the *C* = 0.1 setting, many of the generated molecules were in different chemical space to that of drug-related patented compounds (Figs. 4b and 5b), increasing accidental generation of unusual scaffolds as drug and resulted in the wide distribution of their QEDs. Taken together with the similarity results presented in the previous section, the use of the *R*_patent_ reward function can generate novel molecules in terms of patents with high drug-likeness.

### Examples of generated molecules

**Fig. 6:**
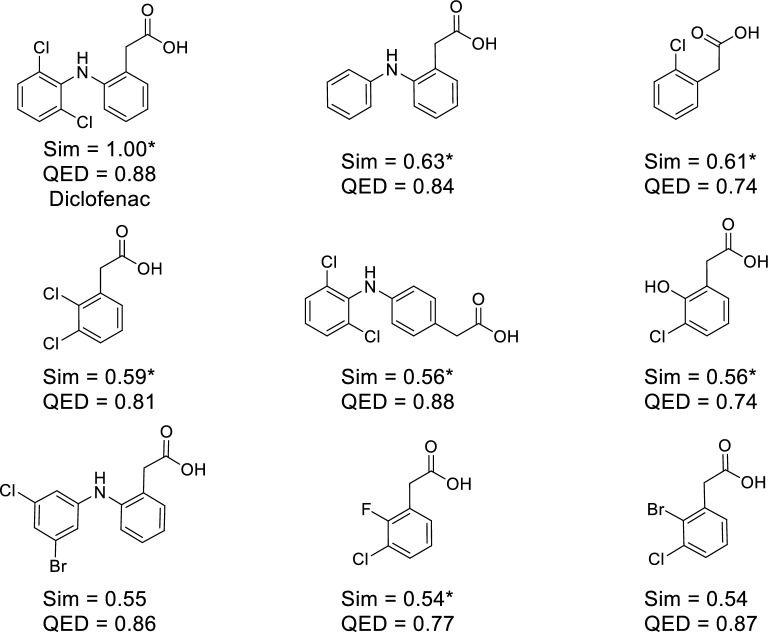
Examples of generated molecules including an approved drug. A generated molecule matched to an approved drug, diclofenac, and generated molecules similar to it are shown. The molecules were generated using the *R*_patent_ reward function. The similarity values to diclofenac and the QED values are indicated below the structures. Compounds registered in the drug-patent DB are marked with an asterisk

Herein, three examples of ChemTS-generating molecules that are structurally similar to approved drugs, diclofenac, baricitinib, and brexpiprazole, are discussed. These molecules have a similarity of ≥ 0.5 to the approved drugs that are not included in the training data of the patent RNN model. They were generated by ChemTS using the patent RNN model, *R*_patent_ reward function, and *C* = 0.4—the conditions under which most molecules were matched to the drug-patent DB compounds (see Fig. 3b). Diclofenac was generated using ChemTS and six of the eight generated diclofenac derivatives were included in the drug-patent DB compounds (Fig. 6).

**Fig. 7:**
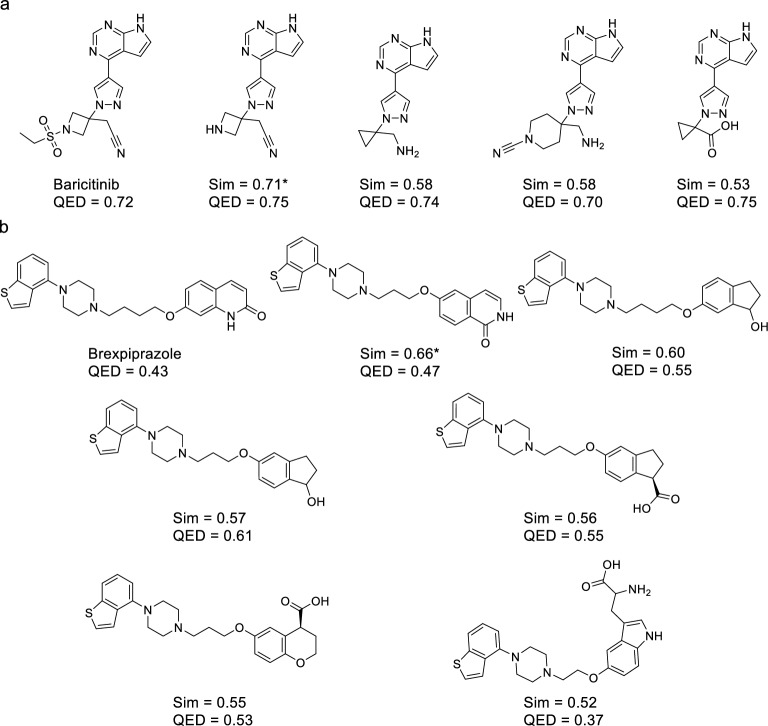
Examples of generated molecules similar to approved drugs. The generated molecules similar to approved drugs, **a** baricitinib and **b** brexpiprazole, are shown with their similarity and QED values. The molecules were generated using the *R*_patent_ reward function. Patented compounds are marked with an asterisk

Baricitinib was not generated using ChemTS, but four baricitinib analogs were generated and one of them was patented (Fig. 7a). Similarly, brexpiprazole was not generated by ChemTS, but six brexpiprazole derivatives were generated and one of them was patented (Fig. 7b). ChemTS can generate molecules similar to approved drugs, but the percentage of generated molecules that are covered by patents vary from case to case (Fig. 7). Regarding drug-likeness, the QED values of the structural analogs of approved drugs generated by ChemTS was case-dependent. The QED values of diclofenac, baricitinib and their derivatives were high (Figs. 6 and 7a). The QED of brexpiprazole analogs was not high; however, most of their QED values were higher than that of brexpiprazole (Fig. 7b). These results suggest that the generative AI developed in this study using *R*_patent_ reward functions can generate non-patented molecules with favorable drug-likeness properties.

## Conclusion

A generative AI that constructs molecules by considering their patentability was developed. To consider patentability, two reward functions, *R*_patent_ and *R*_not-patent_, were defined utilizing a method to determine if the generated molecules are compounds in drug-related patents. The compounds in drug-related patents were extracted from open data and stored in the drug-patent DB, which were also used as the training data of the generative AI (ChemTS). ChemTS, with a drug-related patent RNN and *R*_patent_ reward function, enables molecule generation with the consideration of patentability. Results showed that compounds structurally similar to the approved drugs could be generated without being included in the drug-patent DB. By changing the drug-patent DB used in this study to a database of patented compounds in specific fields, such as agriculture and organic materials, one can use the model for molecular generation in other fields. However, this study only considered the presence of the compounds in the drug-patent DB and not if they are patentable in the true sense of the word. Despite these limitations, the patent-aware molecular generation method developed in this study, in combination with activity and ADMET predictions, is expected to improve drug discovery capabilities through multi-objective optimizations that account for patentability.

### Supplementary Information

**Additional file 1.** Supplemental figures and method.

**Additional file 2.** Substructures that are not appropriate to be learned for RNN.

**Additional file 3.** SMILES dataset used for training the patent RNN.

## Data Availability

The data used in this study were retrieved from the publicly available databases, SureChEMBL and Google Patents Public Datasets. SQL codes for retrieving IPC/CPC information from Google Patents Public Datasets are described in Additional file 1: Method S1. An SQLite database file of the drug-patent DB is downloadable at 10.5281/zenodo.8303450. The training data for the patent RNN are available in Additional file 3.

## Abbreviations

AI: Artificial intelligence
ADMET: Absorption, distribution, metabolism, excretion, and toxicity
CPC: Cooperative Patent Classification
DB: Database
IPC: International Patent Classification
LSH: Locality-sensitive hashing
MCTS: Monte Carlo tree search
MHFP: MinHash fingerprint
OCR: Optical character recognition
QED: Quantitative estimate of drug-likeness
RNN: Recurrent neural network
UMAP: Uniform manifold approximation and projection
USPTO: United States Patent and Trademark Office
