# Supplementary material for: AI-driven molecular generation of not-patented pharmaceutical compounds using world open patent data
Source: J Cheminform. 2023 Dec 13;15:120. doi: 10.1186/s13321-023-00791-z (PMC10716930; doi:10.1186/s13321-023-00791-z)
Supplement: Supplementary file 1 — Additional file 1. Supplemental figures and method. [file 13321_2023_791_MOESM1_ESM.pdf]

Supplementary Information

AI-driven molecular generation of not-patented pharmaceutical compounds using world open patent data

Yugo Shimizu[1,2], Masateru Ohta[1], Shoichi Ishida[3], Kei Terayama[3], Masanori Osawa[2], Teruki Honma[4], Kazuyoshi Ikeda[1,2*]

*Correspondence: Kazuyoshi Ikeda (ikeda-kz@pha.keio.ac.jp)

[1] HPC- and AI-driven Drug Development Platform Division, RIKEN Center for Computational Science, 1-7-22 Suehiro-cho, Tsurumi-ku, Yokohama City, Kanagawa, 230-0045, Japan.
[2] Division of Physics for Life Functions, Keio University Faculty of Pharmacy, 1-5-30 Shibakoen, Minato-ku, Tokyo 105-8512, Japan.
[3] Graduate School of Medical Life Science, Yokohama City University, 1-7-29 Suehiro-cho, Tsurumi-ku, Yokohama City, Kanagawa, 230-0045, Japan.
[4] RIKEN Center for Biosystems Dynamics Research, 1-7-22 Suehiro-cho, Tsurumi-ku, Yokohama City, Kanagawa, 230-0045, Japan.

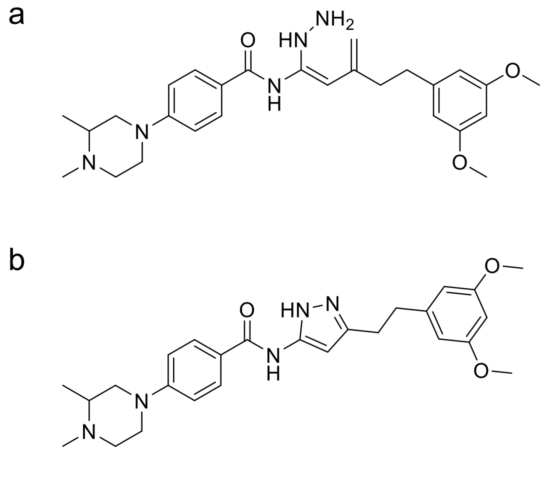

Fig. S1 An example of incorrect registration of chemical structure in SureChEMBL. A SureChEMBL registered structure, SCHEMBL13574165 (a), and the original structure of the chemical (b). The pyrazole ring is broken in SCHEMBL13574165.

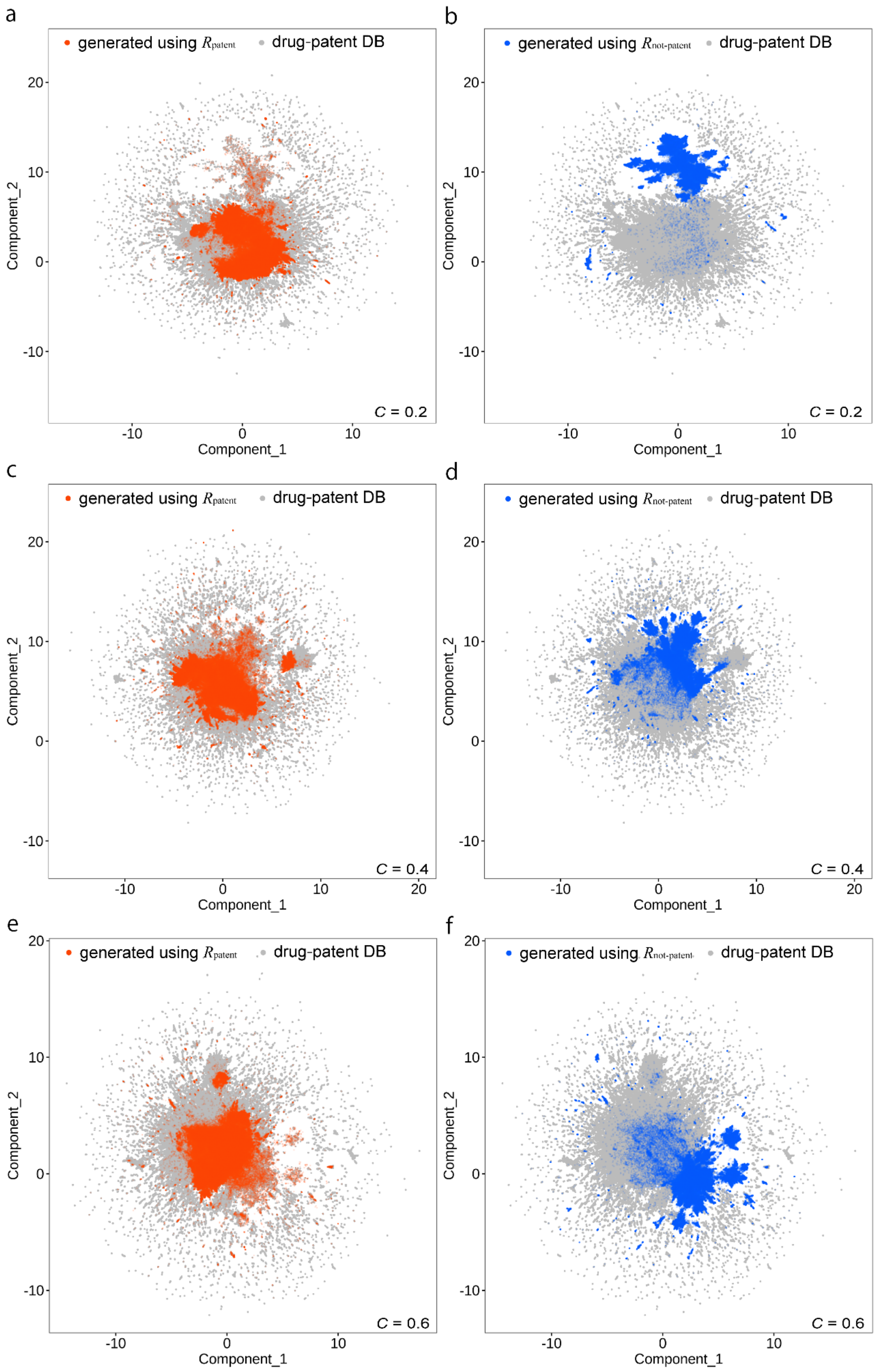
a
generated using R_{patent}
drug-patent DB
C = 0.2
Component_1
Component_2
b
generated using $R_{not-patent}$
drug-patent DB
C = 0.2
Component_1
Component_2
c
generated using R_{patent}
drug-patent DB
C = 0.4
Component_1
Component_2
d
generated using $R_{not-patent}$
drug-patent DB
C = 0.4
Component_1
Component_2
e
generated using R_{patent}
drug-patent DB
C = 0.6
Component_1
Component_2
f
generated using $R_{not-patent}$
drug-patent DB
C = 0.6
Component_1
Component_2

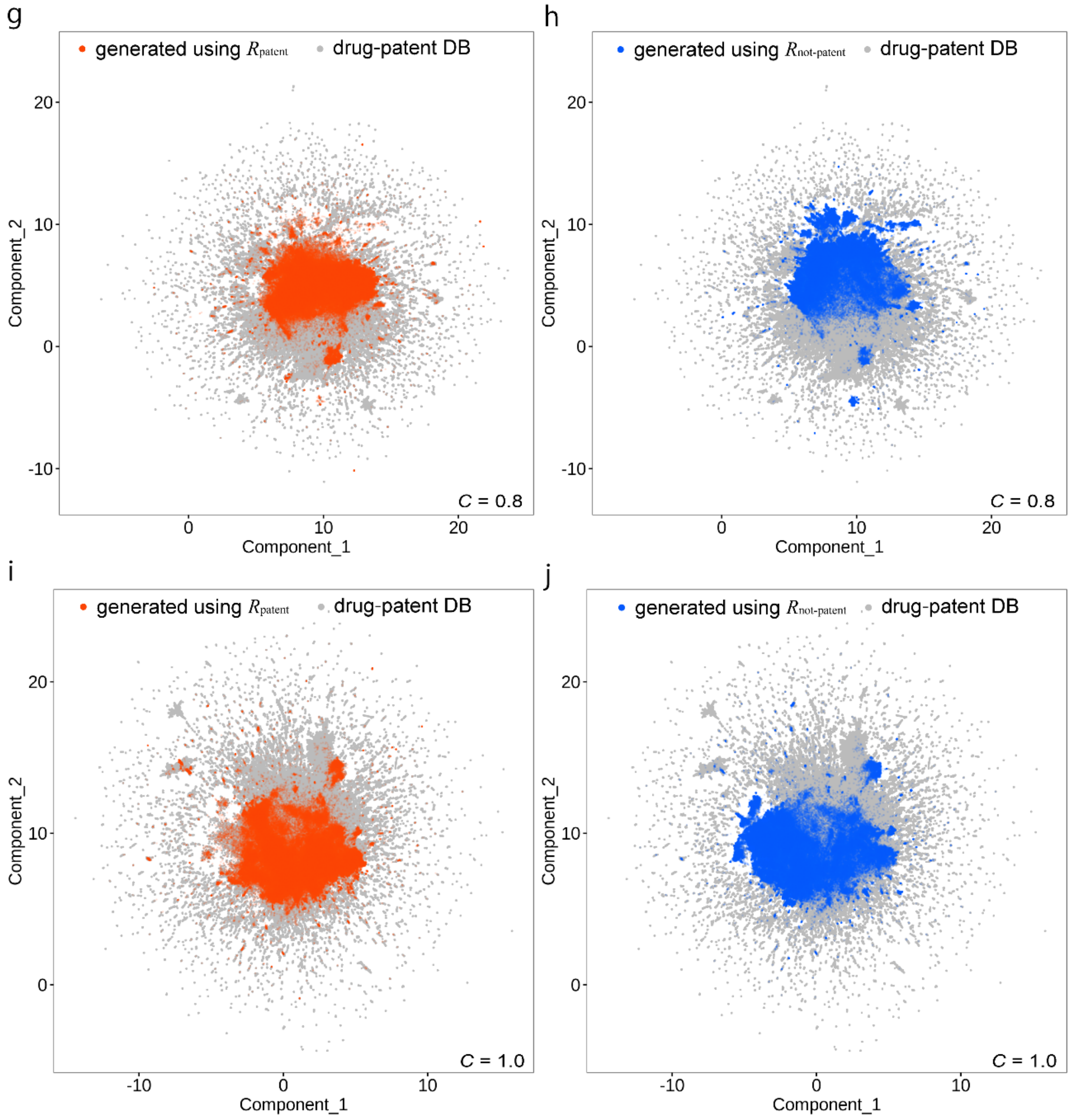

Fig. S2 Chemical space of generated molecules using R_{patent} and $R_{\text{not-patent}}$ as reward functions and the drug-patent DB compounds. Molecules generated using R_{patent} and $R_{\text{not-patent}}$ as reward functions in each *C* setting (0.2, 0.4, 0.6, 0.8, and, 1.0) were compared with 500,000 drug-patent DB compounds. The generated molecules using the R_{patent} and $R_{\text{not-patent}}$ rewards were shown in orange (a, c, e, g, and, i) and blue (b, d, f, h, and, j), respectively, and the drug-patent DB compounds were colored gray in the background. The chemical space was visualized using UMAP.

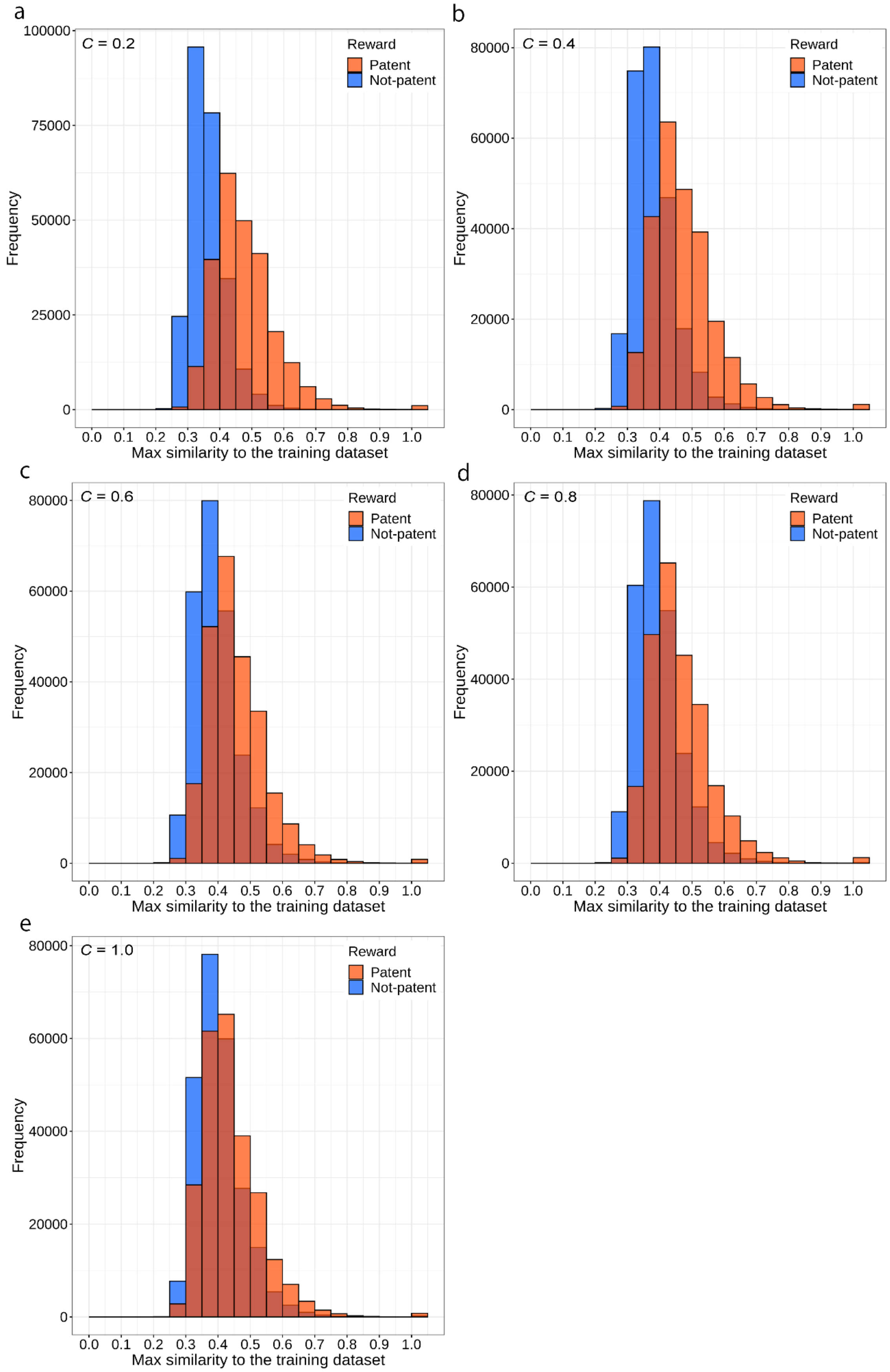

a
C = 0.2
Reward
Patent
Not-patent
Frequency
100000
75000
50000
25000
0
0.0 0.1 0.2 0.3 0.4 0.5 0.6 0.7 0.8 0.9 1.0
Max similarity to the training dataset
b
C = 0.4
80000
60000
40000
20000
c
C = 0.6
d
C = 0.8
e
C = 1.0

Fig. S3 Frequency of structural similarities of generated molecules against the training data. Maximum similarities of generated molecules using the R_{patent} (orange) and $R_{\text{not-patent}}$ (blue) rewards to the 247,738 drug-patent DB compounds used as training data of the patent RNN were plotted as histogram. Molecular generation was performed at $C = 0.2, 0.4, 0.6, 0.8$, and 1.0 (a, b, c, d, and e, respectively).

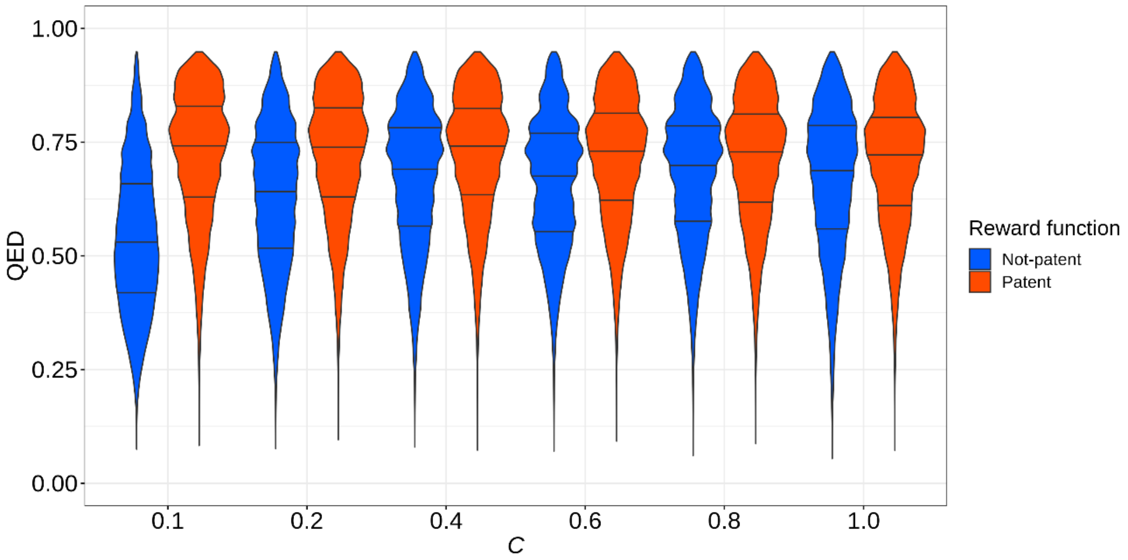

Fig. S4 Violin plot of QED values of the molecules generated using the R_{patent} and $R_{\text{not-patent}}$ reward functions. Horizontal lines in the violin plot indicate the quartile points.

Method S1 SQL codes to retrieve drug-related patents from Google Patents Public Datasets.

For IPC:

```
SELECT
  DISTINCT pubs.publication_number, ipc.code
FROM
  `patents-public-data.patents.publications` AS pubs,
  UNNEST(ipc) AS ipc
WHERE
  (ipc.code LIKE "A61K%" OR ipc.code LIKE "A61P%")
  AND (publication_number LIKE "US%"
    OR publication_number LIKE "EP%"
    OR publication_number LIKE "JP%"
    OR publication_number LIKE "WO%")
```

For CPC:

```
SELECT
  DISTINCT pubs.publication_number, cpc.code
FROM
  `patents-public-data.patents.publications` AS pubs,
  UNNEST(cpc) AS cpc
WHERE
  (cpc.code LIKE "A61K%" OR cpc.code LIKE "A61P%")
  AND (publication_number LIKE "US%"
    OR publication_number LIKE "EP%"
    OR publication_number LIKE "JP%"
    OR publication_number LIKE "WO%")
```
